# Changes in prevalence, and factors associated with tobacco use among Bangladeshi school students: evidence from two nationally representative surveys

**DOI:** 10.1186/s12889-021-10623-0

**Published:** 2021-03-23

**Authors:** Tanvir Ahammed, Nasar U. Ahmed, Md Jamal Uddin

**Affiliations:** 1 Department of Statistics, Shahjalal University of Science and Technology, Sylhet, 3114 Bangladesh; 2 Department of Epidemiology, Robert Stempel College of Public Health & Social Work, Florida International University, Miami, USA

**Keywords:** Adolescents, Bangladesh, Global youth tobacco survey, School, Smoking prevalence, Tobacco use

## Abstract

**Background:**

Globally, tobacco kills more than nine million people per year. Annually in Bangladesh, smoking accounts for 1.2 million illnesses and over one hundred fifty thousand deaths. Worldwide, about one out of five school students smoke tobacco, and this problem is also growing significantly in Bangladesh. There is a need to address this problem. However, to the best of knowledge, no published study has been evaluated the changes in factors associated with tobacco use over time among Bangladeshi adolescent students using large, nationally representative comparable surveys. Our objective was to identify the factors associated with tobacco use among school going students, examine any changes in them over time, and explore policy options based on national surveys.

**Methods:**

We analysed the data from the 2007 and the 2013 Global Youth Tobacco Survey (GYTS), a school-based survey targeting adolescents age 13–15 years (7th–9th grade), developed by the World Health Organization (WHO) and the Centres for Disease Control and Prevention (CDC). The samples were selected based on a two-stage cluster sample design. The data were collected in school classes using a self-administered anonymous survey. We applied chi-square tests and survey logistic regression models for analysing the data.

**Results:**

Overall tobacco usage significantly declined from 8.4 to 6.9% over six years. The prevalence of tobacco use decreased among females (5.22 to 2.84%), those who received anti-tobacco messages (8.93 to 7.24%) and because of age restriction could not buy tobacco products (18.86 to 15.78%). Compared with the female, the odds of overall tobacco smoking among male students was 1.97 (CI: 0.99–3.92) in the year 2007 and it increased (OR = 3.07; CI: 1.56–6.04) in the year 2013. Moreover, the odds of smoking among those exposed to tobacco smoke had increased (OR = 3.26; CI: 1.46–7.29 vs 5.43; CI: 1.63–18.07) from 2007 to 2013.

**Conclusion:**

There was a decline in tobacco use, especially among female students. Male students were higher tobacco user. It appeared anti-smoking campaign and age restriction policies were working.

**Supplementary Information:**

The online version contains supplementary material available at 10.1186/s12889-021-10623-0.

## Background

Tobacco is the only legal drug that kills too many of its users when explicitly used as instructed by its manufacturers [1]. World Health Organization (WHO) estimated eight million deaths annually, and many of these deaths occurred prematurely across the world because of tobacco use [2]. Additionally, second-hand smoking is responsible for 1.2 million deaths per year [2]. Tobacco-related mortality obstructs the social and economic advancement of a country. Untimely death from tobacco decreases the living standards of a family, raises the healthcare cost and hampers the financial condition [2]. As nearly only 20% of the world’s 1.1 billion smokers living in high-income countries [2], Southeast Asia is a high-risk region and experiences 1.2 million tobacco- attributable deaths per annum [3].

Adolescents (age group 10–19 years) make up the majority of the population in South East Asian countries, and they are particularly vulnerable to tobacco use [4, 5]. As Bangladesh is one of the top ten countries with China, India, Indonesia, Russia, the USA, Japan, Brazil, Germany, and Turkey, where the world’s two-thirds of smokers live [6], adolescent tobacco use is also a growing problem for Bangladesh [7, 8]. According to the Global Burden of Disease Study, in 2019, tobacco kills about 157,862 people, which is about 19% of total deaths [9], and there are 1.2 million tobacco-related diseases each year in Bangladesh [10]. A ten-year prospective study of twenty thousand adult participants in Bangladesh has revealed that smoking was responsible for 25% of men’s death and 7.6% of women’s death [11]. It is a clear indication that Bangladesh has a large number of tobacco-related deaths and illnesses that warrants national attention to this enormous problem.

Trends in smoking among adolescents have been increasing across the world [5]. Globally, 21% of school students smoked tobacco [12]. According to the 2013 Global Youth Tobacco Survey (GYTS), 3% of 7-9th grade students were tobacco users, and 2% were cigarette smokers in Bangladesh [13]. However, Bangladesh’s situation was not the worst compared to other South Asian countries. For example, in India, Nepal, Maldives, and Pakistan, the prevalence of smoking among secondary school students was calculated to be at 4.4, 6.9, 4.3, and 3.3%, respectively [12].

In developing countries, over 50% of teenagers who begin smoking at a young age become habitual users [14]. Moreover, research showed that most smokers start smoking before they reach adulthood [15, 16]. Adolescents, in fact, are more likely to develop a nicotine addiction [17], and the majority of adolescents who use tobacco on a daily basis continue to use it later in life. For example, although only 5% of adolescent smokers believe they are still smoking after five years, 75% still smoke after eight years [18]. The probability of contracting tobacco-related diseases is proportional to the time tobacco is used [15]. Some studies in Bangladesh found that smoking is a probable cause of hypertension, coronary artery disease, and acute myocardial infarction in young people [19–21]. One out of three young people who start smoking will eventually face an adverse outcome because of tobacco-related diseases [22]. Therefore, adolescence is the most critical phase for tobacco use initiation, and adolescent smokers are at higher risk for the adverse effect of tobacco use later in life.

The reasons for such prevalent usages, particularly among boys, are thought to reflect tobacco companies’ strategic marketing approach and ineffective execution of tobacco control policies making tobacco products more accessible and affordable to youth. Besides, parental smoking, the best friend’s smoking behaviour, peer pressure, and achieving a poor grade at school influence tobacco use among adolescents [23–27]. Previous studies have also reported a higher prevalence of smoking among adolescents if their favourite film star smokes [28] and if they have sufficient pocket money. Furthermore, tobacco contents in movies are portrayed as societal support for tobacco consumption [29]. There is a paucity of information for adolescent smoking in Bangladesh [16].

In Bangladesh, there are well-established tobacco prevention laws and initiatives, such as currently, there is a national tobacco control legislation named “The Smoking and Tobacco Products Usage (Control) Rule, 2015”, signed by the President of the People’s Republic of Bangladesh on 12 March 2015. It has rectified the Smoking and Tobacco Products Usage (Control) Act, 2005 to correct certain clauses [30]. These initiatives have ensured that tobacco merchandise is priced and taxed appropriately, that smoking is prohibited in public places, that the selling of tobacco products to and by minors is prohibited, that tobacco products are packaged and labelled properly, and prohibiting tobacco advertisements and promotions. These initiatives are combined with increasing awareness through education, training, communication, and research and partnership building to control tobacco use [7].

Despite the initiatives to regulate tobacco use in Bangladesh, adolescent tobacco use’s prevalence rate is still unsatisfactory [31]. While a lot of studies have been conducted on developed countries to look at adolescent tobacco use and its factors, those studies’ results are not sufficient for developing countries, particularly Bangladesh. Because of a lack of national-level information, we do not have sufficient knowledge of the effect of the antitobacco laws on the adolescents smoking trends in Bangladesh.

Considering the long-term adverse health effects of adolescents tobacco use and its long-term effect on the country’s economic growth, we analysed two available datasets from the Global Youth Tobacco Survey (GYTS) during 2007 and 2013, intending to identify the factors associated with adolescents tobacco use in Bangladesh and to examine changes in these factors between the GYTS 2007 and 2013 surveys and to explore the policy relevance.

## Methods

### Data source

We extracted the data from the 2007 and 2013 Bangladesh Global Youth Tobacco Surveys (GYTS). The surveys were school-based, nationwide representative, cross-sectional surveys of secondary school students. Briefly, a two-stage cluster sampling design was used to construct nationally representative samples. All the secondary school students of grades 7 to 10 in 2007 and grades 7 to 9 in 2013, i.e., 11–17 years students, were included in the sampling frame. In the 2007 national sample of GYTS, 3113 Bangladeshi students were selected from 52 schools, while 50 schools with a total of 3245 students participated in the GYTS 2013 survey. The GYTS survey report for Bangladesh contains detailed data collection procedures [7, 32].

Demographics, tobacco usage, tobacco use cessation, exposure to tobacco, media messages and ads, accessibility to and affordability of tobacco products, and awareness and attitudes against tobacco products were all covered by the GYTS (self-administered in classrooms) questionnaires. Students were asked to choose a single answer from a list of multiple-choice answers for each question [8, 31].

### Ethics approval and consent to participate

Bangladesh Medical Research Council Ethical Review Committee reviewed and approved the GYTS study protocol. Participant anonymity and confidentiality was maintained throughout the GYTS survey procedure. School authorities gave written permissions, and all respondents provided signed letters of informed consent before data collection [7, 31].

### Outcome variables

The outcome variables (dependent variables) were the current tobacco use (from now on, we will write tobacco use instead of current tobacco use), smoking tobacco use, and smokeless tobacco use. Participants were asked whether they had smoked a cigarette, smoked a bidi or chewed tobacco at least once in the previous 30 days prior to the survey. The “yes” / “no,” answers constitute the dependent variables used to study the behaviour of tobacco use.

### Factors associated with outcome variables

From the wide range of GYTS factors (independent variables), some important factors were selected as factors associated with tobacco use, which were age, sex, and grade of an adolescent, exposure to second-hand smoking, the amount of pocket money received (adjusted for inflation from 2007 to 2013), average costs of a pack cigarettes (adjusted for inflation), the selling of tobacco products free of charges by vendors, watching advertisements and marketing campaigns in print and electronic media, learned in the classes about the risk of smoking as a school lesson, the vendor refuses to sell cigarettes because of age, and favoured banning smoking at public places. Adolescents’ age was categorised as ‘younger than 14 years’, ‘equal to 14 years’, and ‘older than 14 years.’ Similarly, a student’s grade was classified as ‘seventh’, ‘eighth’, and ‘ninth and above.’ The students were asked how many days anyone had smoked in their presence. Students with a response of more than 0 days were considered as exposed to tobacco use. In 2007, adjusted for inflation from 2007 to 2013, the average monthly allowance (pocket money) was categorised as ‘spend less than or equal to 160 TK’ and ‘spend more than 160 TK’. However, in the 2013 survey, the variable value fell in the three different categories, i.e., ‘spend less than or equal to 400 TK’, ‘spend between 400 and 800 TK’, and ‘spend more than or equal to 800 TK’. Similarly, after adjusting for inflation, in 2007, the cost of a pack of cigarettes was categorised as ‘less than 64 TK’, ‘more than or equal to 64 TK’ whereas in 2013, the categories were: ‘less than or equal to 75 TK’, ‘between 76 to 200 TK’, and ‘more than 200 TK’. The selection of the factors associated with tobacco use was guided by related studies in the literature related to adolescent tobacco use [8, 13, 33, 34].

### Statistical analysis

We analysed each dataset separately to identify, compare and contrast the factors associated with tobacco use between two surveys and then used pool analysis by combing the 2007 and 2013 GYTS datasets. In the pooled analysis, we adjusted the regression models for the time (year) using surveys’ years as an independent variable in the model. A descriptive analysis was undertaken to characterise the sample. For each student record, a weighting factor is added to account for nonresponse and variation in the selection probability at the school, grade, and student levels. Then the univariate analysis between current tobacco users and the potential factors associated with tobacco use was done. According to Hosmer and Lemeshow’s purposeful covariates selection [35, 36], factors with *P*-value less than 0.25 in the univariate analysis was elected for the multiple binary survey logistic regression (say, Model I). Only factors with *P* ≤ 0.05 in the previous model were included in the final model (Model II). We used the ‘Survey Logistic’ procedure in SAS to perform the analysis for multi-stage cluster sampling used in GYTS. In order to show how likely the interest group to be a tobacco user relative to the reference group, the odds ratio (OR) with 95% confidence interval (CI) in favour of current tobacco use were calculated for the coefficient of each of the factors. Then we applied the receiver operating characteristic curve (ROC) analyses to evaluate the models’ performance by computing the area under the curve (AUC). A ROC curve is a graph that plots 1 – specificity on the horizontal axis and sensitivity on the vertical axis where the AUC indicates the model accuracy. The AUC value lies between 0 to 1, where 0 denotes a completely inaccurate classifier, and 1 indicates  a perfectly accurate classifier, and an AUC of 0.5 suggests no discriminatory ability of the model. Simply, the closer the AUC value is to 1, the better the model [37]. Finally, we performed a forest plot representing the odds ratio and 95% confidence intervals of the significant factors from the pooled dataset.

## Results

Among 3113 selected students in 2007, 49.53% (*n* = 1542) students were male whereas, in 2013, there were 41.08% (*n* = 1333) male students and most of the students were from 9th grade and above (2007: 55.57%; vs 2013: 38.52%). More students in 2007 (79.57%) favoured banning smoking in public places than in 2013 (62.68%). Moreover, in 2013, the number of students who were exposed to tobacco smoke was increased from 48.02 to 68.84%.

### Prevalence and epidemiology of tobacco use

The prevalence of overall tobacco usages significantly (*P* = 0.001) declined from 8.4% (2,696,281 users) in 2007 to 6.9% (2,213,560 users) in 2013 resulted in about 0.4 million fewer tobacco users in that age group of students. Similarly, the prevalence of smoking tobacco usages declined from 4.2% (1,348,141 users) to 2.94% (943,169 users) and the prevalence of smokeless tobacco usages declined from 5.29% (1,698,015 users) to 4.56% (1,462,874 users).

Table 1 presents the prevalence of the overall tobacco use of the two surveys according to the selected factors associated with tobacco use among Bangladeshi students. In both survey years, the prevalence of male smokers was higher, and the gap increased in 2013 (5.18% vs 6.69%). Regarding age and grade, the pattern of the tobacco use prevalence was the same in the two surveys: there was a higher prevalence for older and senior grade students. On average, adolescents received more pocket money in 2013 than in 2007, and in both the surveys, there was a higher prevalence of tobacco use among the adolescents who received more money. For example, in 2007, 26.59% of those who received more than 160 TK per month were tobacco users, whereas only 7.02% of those who received less than or equal to 160 TK were tobacco users. The prevalence of tobacco use was increasingly lower among adolescents who favoured banning smoking at public places versus those against it (2007: 7.79% vs 8.51%; 2013: 5.31% vs 9.74%) and who were taught about the danger of tobacco in class versus those who did not (2007: 6.65% vs 7.96%; 2013: 6.89% vs 7.35%). Furthermore, the prevalence of tobacco use was higher among participants who observed advertisements and promotional campaigns in newspapers or magazines (2007: 9.00% vs 5.97%; 2013: 8.76% vs 6.01%) and were exposed to tobacco smoke than those who did not (2007: 12.56% vs 3.42%; 2013: 9.35% vs 1.29%).

**Table 1:** Comparison of the prevalence of current tobacco use in Bangladeshi students between GYTS (2007) and GYTS (2013) survey

Covariates	Survey Year 2007			Survey Year 2013		
	Overall Tobacco Use					
	No	Yes	*P* Value	No	Yes	*P* Value
	Unweighted Frequency (Weighted Percent)	Unweighted Frequency (Weighted Percent)		Unweighted Frequency (Weighted Percent)	Unweighted Frequency (Weighted Percent)	
Sex			<.001			<.001
Male	1325 (89.60)	217 (10.40)		1208 (90.83)	125 (9.17)	
Female	1339 (94.78)	103 (5.22)		1679 (97.16)	97 (2.84)	
Age			<.001			<.001
Younger than 14 years	989 (93.86)	131 (6.14)		1423 (95.05)	84 (4.95)	
Equal to 14 years	828 (93.32)	68 (6.68)		1027 (92.26)	77 (7.74)	
Older than 14 years	891 (86.81)	136 (13.19)		434 (85.47)	61 (14.53)	
Grade			0.007			0.667
7th	553 (94.10)	57 (5.90)		899 (94.88)	64 (5.12)	
8th	657 (91.79)	64 (8.21)		832 (92.61)	62 (7.39)	
9th or above	1510 (89.75)	220 (10.25)		1155 (91.39)	95 (8.61)	
Average pocket money (per month)^a^			<.001			<.001
Spend less than or equal to 160 TK	2391 (92.98)	227 (7.02)				
Spend more than 160 TK	303 (73.41)	102 (26.59)				
Spend less than or equal to 400 TK				2649 (93.96)	159 (6.04)	
Spend between 400 and 800 TK				157 (76.34)	51 (23.66)	
Spend more than or equal to 800 TK				66 (88.73)	12 (11.27)	
Aware of the effect of passive smoking			<.001			<.001
Yes	2395 (91.97)	250 (8.03)		2650 (93.31)	180 (6.69)	
No	301 (88.54)	86 (11.46)		226 (95.61)	35 (4.39)	
Favoured banning smoking at public places			<.001			0.003
Yes	2244 (92.21)	233 (7.79)		1909 (94.69)	125 (5.31)	
No	436 (91.49)	79 (8.51)		958 (90.26)	95 (9.74)	
Refused by the tobacco vendor for age			0.006			0.001
Yes	170 (81.14)	70 (18.86)		127 (84.22)	25 (15.78)	
No	250 (90.30)	59 (9.70)		24 (57.28)	16 (42.72)	
Average cost per packet cigarette (20 cigarettes)^a^			0.021			<.001
Less than 64TK	10 (71.14)	17 (28.86)				
More than or equal to 64 TK	5 (45.14)	34 (54.86)				
Less than or equal to 75 TK				363 (89.08)	42 (10.92)	
Between 76 to 200 TK				132 (73.76)	70 (26.24)	
More than 200 Tk				1891 (95.40)	83 (4.60)	
Received anti-tobacco message			0.205			0.428
Yes	2420 (91.07)	285 (8.93)		2150 (92.76)	181 (7.24)	
No	75 (94.60)	13 (5.40)		275 (93.09)	19 (6.91)	
Health warning on tobacco products						<.001
Yes				2134 (92.21)	195 (7.79)	
No				737 (96.55)	27 (3.45)	
Being offered free tobacco products			<.001			0.033
Yes	444 (84.10)	134 (15.90)		73 (95.28)	11 (4.72)	
No	2202 (93.41)	187 (6.59)		2790 (93.02)	210 (6.98)	
Noticed anyone smoking in the school						<.001
Yes				1004 (90.05)	138 (9.95)	
No				1870 (95.64)	77 (4.36)	
Taught about danger of tobacco in class			0.005			0.558
Yes	1288 (93.35)	125 (6.65)		1640 (93.11)	129 (6.89)	
No	1007 (92.04)	141 (7.96)		789 (92.65)	68 (7.35)	
Tobacco advertisement			0.274			0.104
Yes	1739 (91.00)	221 (9.00)		1969 (91.24)	177 (8.76)	
No	85 (94.03)	7 (5.97)		261 (93.99)	15 (6.01)	
Exposed to tobacco smoke			<.001			<.001
Yes	1255 (87.44)	240 (12.56)		2027 (90.65)	207 (9.35)	
No	1461 (96.58)	96 (3.42)		846 (98.71)	15 (1.29)	

*GYTS* Global Youth Tobacco Survey. TK: Bangladeshi currency (1 USD ≈ 85 TK)

^a^ Information of ‘average pocket money (per month)’ and ‘average cost per packet cigarette (20 cigarettes)’ were collected in different categories in 2007 and 2013 surveys and could not be combined in the same categories

### Logistic regression

The univariate logistic regression results and binary logistic regression results from the model I was showed in Table 2 and Additional file 1, respectively.

**Table 2:** Factors associated with overall tobacco use among adolescents for both survey years by univariate logistic regression analysis

Covariates	Survey Year 2007		Survey Year 2013	
	Odds Ratio (95% CI)	*P*-value	Odds Ratio (95% CI)	*P*-value
Sex				
Female	1		1	
Male	2.11 [1.09–4.07]	0.028	3.45 [1.73–6.90]	0.001
Age				
Younger than 14 years	1		1	
Equal to 14 years	1.09 [0.69–1.73]	0.689	1.61 [0.90–2.87]	0.102
Older than 14 years	2.32 [1.30–4.14]	0.006	3.26 [1.48–7.18]	0.005
Grade				
7th	1		1	
8th	1.43 [0.58–3.50]	0.422	1.48 [0.75–2.90]	0.246
9th or above	1.82 [0.74–4.51]	0.185	1.75 [0.55–5.57]	0.334
Average pocket money (per month)				
Less than or equal to 160 TK	1			
More than 160 TK	4.80 [2.63–8.78]	<.0001		
Spend less than or equal to 400 TK			1	
Spend between 400 and 800 TK			4.82 [0.89–26.10]	0.067
Spend more than or equal to 800 TK			1.98 [0.28–13.71]	0.477
Aware of the effect of passive smoking				
Yes	1		1	
No	1.48 [0.72–3.05]	0.273	0.641 [0.22–1.84]	0.393
Favoured banning smoking at public places				
Yes	1		1	
No	1.10 [0.52–2.31]	0.793	1.92 [1.06–3.50]	0.034
Refused by the tobacco vendor for age				
Yes	2.16 [1.04–4.51]	0.040	0.25 [0.06–1.06]	0.060
No	1			
Average cost per packet cigarette (20 cigarettes)				
Less than 64TK	1			
More than or equal to 64 TK	3.00 [0.94–9.54]	0.061		
More than 200 Tk			1	
Less than or equal to 75 TK			2.54 [0.79–8.17]	0.112
Between 76 to 200 TK			7.38 [3.22–16.92]	<.001
Received anti-tobacco message				
Yes	1.72 [0.61–4.88]	0.295	1.05 [0.31–3.62]	0.934
No	1		1	
Health warning on tobacco products				
Yes			2.37 [1.14–4.91]	0.022
No			1	
Noticed anyone smoking in the school				
Yes			1	
No			2.42 [1.45–3.95]	0.001
Being offered free tobacco products				
Yes	2.68 [1.46–4.91]	0.003	0.66 [0.14–3.12]	0.588
No	1		1	
Taught about danger of tobacco in class				
Yes	1		1	
No	1.21 [0.78–1.90]	0.380	1.07 [0.46–2.50]	0.868
Tobacco advertisement				
Yes	1.56 [0.57–4.29]	0.376	1.50 [0.81–2.80]	0.191
No	1		1	
Exposed to tobacco smoke				
Yes	4.06 [1.90–8.66]	0.001	7.91 [2.74–22.90]	0.001
No	1		1	

*CI* Confidence Interval. TK: Bangladeshi currency (1 USD ≈ 85 TK)

The binary logistic regression results showed (Table 3) that the odds ratio of tobacco use among boys increased between the two surveys (OR = 1.97; CI: 0.99–3.92 vs OR = 3.07; CI: 1.56–6.04). We found a similar increasing pattern among the students exposed to tobacco smoke (OR = 3.26 vs OR = 5.43). In addition to these factors: the amount of average pocket money received was found significant (*P* < 0.05) for 2007 survey data, whereas in 2013, age, opinion on banning smoking at public places, per-packet cigarette’s cost, and noticed anyone smoking in the school were found significant (*P *< 0.05).

**Table 3:** Factors associated with overall, smoking, and smokeless tobacco use among adolescents for both survey years (Model II) by binary logistic regression analysis

Covariates	Survey Year 2007		Survey Year 2013		Survey Year 2007		Survey Year 2013		Survey Year 2007		Survey Year 2013	
	Overall Tobacco Use^a^				Smoking Tobacco Use^a^				Smokeless Tobacco Use^a^			
	Odds Ratio (95% CI)	*P*-value	Odds Ratio (95% CI)	*P*-value	Odds Ratio (95% CI)	*P*-value	Odds Ratio (95% CI)	*P*-value	Odds Ratio (95% CI)	P-value	Odds Ratio (95% CI)	*P*-value
Sex												
Female	1		1		1						1	
Male	1.97 [0.99–3.92]	0.050	3.07 [1.56–6.04]	0.002	3.89 [1.67–9.05]	0.003					2.59 [1.13–5.92]	0.026
Age												
Younger than 14 years			1		1		1				1	
Equal to 14 years			1.66 [0.89–3.11]	0.109	0.46 [0.17–1.25]	0.123	1.85 [0.63–5.43]	0.249			1.80 [0.67–4.80]	0.231
Older than 14 years			3.94 [2.22–7.01]	<.001	1.52 [0.61–3.75]	0.356	3.76 [1.30–10.90]	0.017			3.47 [1.65–7.30]	0.002
Grade												
7th					1							
8th					4.75 [1.26–18.00]	0.024						
9th or above					6.73 [1.65–27.40]	0.010						
Average pocket money (per month)												
Less than or equal to 160 TK	1				1							
More than 160 TK	4.26 [2.07–8.80]	<.001			6.10 [2.34–15.88]	<.001						
Favoured banning smoking at public places												
Yes			1		1		1					
No			1.80 [1.00–3.24]	0.049	2.54 [1.15–5.63]	0.023	5.58 [2.10–14.81]	0.001				
Average cost per packet cigarette (20 cigarettes)												
More than 200 Tk			1				1					
Less than or equal to 75 TK			1.55 [0.67–3.59]	0.298			11.52 [2.67–49.66]	0.002				
Between 76 to 200 TK			6.62 [3.19–13.71]	<.001			40.63 [11.05–149.4]	<.001				
Noticed anyone smoking in the school												
No			1								1	
Yes			2.19 [1.23–3.91]	0.01							2.68 [1.65–4.37]	<.001
Exposed to tobacco smoke												
Yes	3.26 [1.46–7.29]	0.006	5.43 [1.63–18.07]	0.008	3.41 [1.56–7.47]	0.004	7.45 [1.68–33.11]	0.010	3.36 [1.25–9.03]	0.018	5.36 [1.45–19.86]	0.014
No	1		1		1		1		1		1	

*CI* Confidence Interval. TK: Bangladeshi currency (1 USD ≈ 85 TK)

^a^Dependent variable

We have also explored factors associated with smoking and smokeless tobacco use. For both datasets, older than 14 years (2007 OR = 1.52, CI: 0.61–3.75; vs 2013 OR = 3.76, CI: 1.30–10.90), against banning smoking at a public place (OR = 2.54 CI: 1.15–5.63 and 5.58, CI: 2.10–14.81), and exposure to tobacco use by others (OR = 3.41, CI: 1.56–7.47 and 7.45, CI: 1.68–33.11) were positively associated with smoking tobacco use. However, in addition to these factors, in 2007, male sex (OR = 3.89; CI: 1.67–9.05), higher grade (OR for 8th grade = 4.75, CI: 1.26–18.00; OR for 9th grade or above = 6.73; CI: 1.65–27.40), and the higher amount of received pocket money (OR = 6.10; CI: 2.34–15.88), and in 2013, the lower price of a pack of cigarettes (OR for less than or equal to 75 TK = 11.52, CI: 2.67–49.66; OR for between 76 to 200 TK = 40.63; CI: 11.05–149.40) were significant factors and positively associated with smoking tobacco use (Table 3).

Exposure to tobacco use by others was positively associated with smokeless tobacco use (OR = 3.36, CI: 1.25–9.03) compared to those who were not, was the only significant factor for the 2007 data set. On the other hand, in 2013, male (OR = 2.59, CI: 1.13–5.92), older than 14 years (OR = 3.47, CI: 1.65–7.30), noticed anyone smoking in the school (OR = 2.68, CI: 1.65–4.37), and exposed to smokeless tobacco (OR = 5.36, CI: 1.45–19.86) were positively associated with smokeless tobacco use (Table 3).

ROC curves for both 2007 and 2013 survey data were illustrated in Additional file 2. The higher Area Under the Curve (AUC) for 2013 survey data (AUC = 0.708 vs AUC = 0.793) indicated the better performance of the model at distinguishing between the tobacco user and non-user classes. More precisely, the accuracy of the model using 2013 survey data was 79% and for the model using 2007 survey data was 71% which indicated good fits of the models.

Table 4 represents the odds ratio (with 95% confidence intervals) of significant factors in the pooled datasets. Overall male students (OR = 2.19, 95% CI: 1.35–3.56, *P*-value = 0.002), students older than 14 years (OR = 2.19, 95% CI: 1.26–3.79, P-value = 0.006), against banning smoking (OR = 1.92, 95% CI: 1.19–3.09, P-value = 0.008), received more pocket money (OR for pocket money between 16 to 200 TK: 2.86, 95% CI: 1.62–5.06, P-value < 0.001; OR for pocket money more than 200 TK: 5.91, 95% CI: 1.92–18.19, P-value < 0.003) and exposed to tobacco smoke (OR = 4.18, 95% CI: 2.23–7.82, P-value < 0.001) positively associated with usages of tobacco products. This time the AUC is 0.727 (Additional file 3). Older age and exposure to tobacco use by others had positively associated with smoking and smokeless tobacco use. Moreover, male sex and receiving a higher amount of pocket money were also associated with smoking tobacco use (Table 4). The pooled dataset results showed a similar pattern as found in the 2007 and 2013 datasets separately.

**Table 4:** Factors associated with overall, smoking, and smokeless tobacco use among adolescents using pooled dataset by binary logistic regression analysis

Covariates	Overall Tobacco Use^a^		Smoking Tobacco Use^a^		Smokeless Tobacco Use^a^	
	Odds Ratio (95% CI)	*P*-value	Odds Ratio (95% CI)	*P*-value	Odds Ratio (95% CI)	*P*-value
Sex						
Female	1		1			
Male	2.19 [1.35–3.56]	0.002	3.18 [1.60–6.31]	0.001		
Age						
Younger than 14 years	1		1		1	
Equal to 14 years	1.37 [0.88–2.13]	0.155	1.22 [0.54–2.80]	0.627	1.62 [0.89–2.96]	0.111
Older than 14 years	2.19 [1.26–3.79]	0.006	2.57 [1.20–5.53]	0.017	2.47 [1.29–4.73]	0.007
Average pocket money (per month)						
Less than or equal to 16 TK	1		1			
Between 16 and 200 TK	2.86 [1.62–5.06]	0.001	3.97 [1.51–10.42]	0.006		
More than 200 TK	5.91 [1.92–18.19]	0.003	10.84 [1.53–76.68]	0.018		
Favoured banning smoking at public places						
Yes	1		1			
No	1.92 [1.19–3.09]	0.008	3.77 [1.81–7.85]	0.001		
Exposed to tobacco						
Yes	4.18 [2.23–7.82]	0.002	5.29 [2.55–10.99]	<.001	4.01 [1.87–8.58]	0.001
No	1		1		1	

*CI* Confidence Interval. TK: Bangladeshi currency (1 USD ≈ 85 TK)

^a^Dependent variable

## Discussion

Our study is the first comprehensive evaluations of changes in patterns of tobacco use over time among adolescent students in Bangladesh using large, nationally representative comparable surveys. The surveys were cross-sectional and adolescent school students’ samples were independent. We found that the prevalence rate of tobacco use among Bangladeshi students decreased by more than 1 % point, consistent with the findings of other studies [8, 31, 32]. A declined in tobacco use prevalence between 2007 and 2013 does not necessarily mean a decrease in the number of adolescent tobacco users, given the population growth and changing age composition. However, in 2007, ‘10–19 years’ age group represented 22.5% (32.1 million) of the entire population (142.7 million) of Bangladesh [38, 39] resulted in about 2.7 million estimated tobacco users in that age group. On the other hand, in 2013, 21% (32.08 million) of the entire population (152.8 million) [39, 40] fell in that age group having an estimated 2.2 million tobacco users. We observed similar patterns regarding smoking and smokeless tobacco users. Therefore, it is a clear indication of a decline in the number of adolescent tobacco users. The decrease in prevalence may reflect the effect of the tobacco control policy in Bangladesh [7, 8]. However, the decreasing rate is not adequate to control the problem.

Looking at the characteristics of the users, the percentage of male tobacco users was higher than that of female users in this study, and the difference between the male-female ratio slightly increased between the two surveys (male 10.4% & female 5.2% vs male 9.2% & female 2.8%). As male predominance in the smoking prevalence is not unexpected as it was found in most countries [8, 13]. However, contrary to the previous findings, this study showed that the girls’ user rate is now decreasing [8]. Possible reasons could be the social acceptability and attitudes about smoking vary between males and females, and society frowns upon females’ smoking in male-dominated countries [41–43]. According to the 2007 Bangladesh GYTS survey, 26% believe boys, while only 10% believe girls who smoke have more friends. In addition, 45% of them find boys, and 11% find girls who smoke are more appealing [44]. Female tobacco user, while currently low, may increase and will also require monitoring. Bangladesh has lagged behind other South Asian countries in terms of teaching teenagers about the risks of tobacco use [13]. For school-aged children, the advantages of early education programs have been well established. These initiatives should reflect on the harmful effects of tobacco use.

In line with the previous studies [8, 31], age was not significantly associated with overall tobacco use and smokeless tobacco use in 2007, but in 2013, there was a significant difference in tobacco use for students regarding age. When comparing students aged above 14 years to those younger than 14 years, older students were almost four times more likely to be tobacco users. It indicated that tobacco use is more common among older school students. Thus, prevention and cessation programs need to be initiated for these groups.

The prevalence of overall tobacco use was significantly higher among the adolescents receiving a higher amount of pocket money in 2007 aligns with results from other studies [8, 31]. This could indicate that having spare money is responsible for easier access to tobacco products, and therefore, initiate tobacco using [10]. Besides, similar to previous findings [5], we found a relationship between smoking and overall tobacco use and the lower price of a pack of cigarettes in 2013. Therefore, raising the price of tobacco products would be useful in reducing tobacco use among adolescents. While other factors influence the price of tobacco products, the rise in the excise tax has generally resulted in an equal or more dramatic rise in the price of tobacco products. Therefore, it makes tobacco products less desirable and affordable to adolescents with minimal financial ability and less option for spending available money [45]. For example, a uniform excise tax on average could reduce 27% smoking prevalence among adolescents [5]. In Bangladesh, there were significant rises in prices. However, because of increased pocket money, adolescents can still afford tobacco products [46]. Increasing awareness among guardians about pocket money through different campaigns would be helpful in this regard.

Students in this study were exposed to tobacco smoke everywhere. This smoking-friendly atmosphere exposes them to health risks and encourages them to start consuming tobacco. Consistent with other studies in the literature [8, 33, 34, 47], we also observed significantly higher odds ratios of tobacco use among adolescents exposed to tobacco smoke compared to their counterparts. Exposure to smoking significantly increases the tobacco use risk three to seven times, indicating that the scope and enforcement of smoke-free policies were not enough. Furthermore, consistent with Khan et al.’s findings [31], a significant association was found between smoking and overall tobacco use and students’ negative attitudes towards banning smoking in public places in 2013. To overcome this problem, the Bangladesh government banned smoking in public places through the 2015 Smoking and Tobacco Products Usage (Control) Rule [48]. However, these laws are yet to be strictly enforced. Weak or non-existent enforcement of such legislation results in prevalent tobacco usage in public places. Smoking bans in public places were found to have a major preventive impact on the prevalence of smoking [49]. Therefore, more vigorous implementation of laws and policies and smoking bans in public places is essential. Furthermore, in model I (Additional file 1), we observed that being offered free tobacco products from the vendors was positively associated with overall tobacco use. However, the scenario changed completely in 2013. Maybe this is because, though the current law in Bangladesh prohibits direct tobacco advertisement, the tobacco industries use sneaky ways to advertise their products [31] and maybe trying to create new consumers by offering free products to the non-users rather than existing users. Therefore, it is necessary to intensify the tobacco control acts in Bangladesh. It will be useful to create a National Health Research Institute for assessing the effect of tobacco marketing campaigns. This program will pave the foundations for a greater understanding of tobacco advertisement, promotion, and distribution, as well as their effects on adolescents [50]. Other initiatives, such as age limits in purchasing tobacco products, awareness campaigns about the adverse effects of tobacco use, using dramas and movies to portray unfavourable outcomes, could also play a vital role along with law enforcement.

### Strengths and limitations

This study used data from two nationally representative surveys in Bangladesh. We used several sensitivity analyses using smoking and smokeless tobacco use as outcome variables, and the results were consistent. Furthermore, we used pooled analysis to assess the factors associated with tobacco use. The findings of this study clarify the factors involved in adolescents’ tobacco use and changes in them over time and justify updating policy options. No published study has evaluated this research question. These are the strengths of this study; however, it has some limitations too. First, the results only highlight the school-going adolescent in Bangladesh and do not represent the entire adolescent population. The out of school children may have a much higher prevalence than the surveys captured. Second, these data based on self-reports may be subject to memory bias and intentional misreporting. Although anonymity was emphasized and attempts participants were ensured confidentiality by the data collectors, the participants may have underrepresented their tobacco use behaviour as smoking is not encouraged in Bangladesh. Third, because of monetary inflation, some factors such as pocket money, the average cost of a pack of cigarettes cannot precisely be compared between the two surveys, however, we have converted the value at the 2013 level. Fourth, it has been a while since the two surveys were conducted, and there are maybe more factors to think of, i.e., effects of social media. In addition to that, maybe there are several other factors not included in the survey but influencing tobacco use (e.g., parents’ education and occupation, social status). These factors could offer further insights into tobacco use in school students. Fifth, the data used for the analysis were for 2007 and 2013, which did not reflect the changes intended in the policy shifts due to the Smoking and Using of Tobacco Products (Control) (Amendment) Act, 2013 which contains the improvements to the Smoking and Tobacco Products Usage (Control) Act, 2005. Finally, the nature of the study was cross-sectional, therefore, lack of temporality information prevents the determination of cause-and-effect relationships.

### Recommendations

As the prevalence of tobacco usages among school-going adolescents remains high, it is necessary to periodically assess the prevalence and preventive measures of tobacco use and update the prevention policies based on up-to-date information for Bangladesh. Adults should be more careful about tobacco use in front of adolescents, whether at home or elsewhere and track their adolescents’ use of pocket money. Boys should be given the highest priority in policymaking and strategic planning since they are more prone to tobacco use than girls. Tobacco use by students and staffs should be strictly forbidden by school authorities. Moreover, they should keep tobacco vendors out of their school compounds. Additionally, more barriers to limit tobacco products’ accessibility, fines and punishments for tobacco vendors selling tobacco products free of charge to the adolescents, increases in the tobacco products excise duties, and more substantial public places restrictions on smoking should be geared up to strengthen the existing tobacco control policies for decreasing tobacco use and increasing tobacco cessation among adolescents.

## Conclusions

In Bangladesh, there was a decline in tobacco use among school-going adolescents, especially among female students. It appeared that established tobacco control laws and policies, i.e., anti-smoking campaigns, age restriction policies, were working. However, the prevalence of tobacco use is still high. Controlling adolescent tobacco use may be more effective if a combination of government-social-and home-based anti-smoking strategies and interventions are used.

## Supplementary Information

**Additional file 1.** The odds ratios for factors influencing tobacco use among adolescents for both survey years by binary logistic regression analysis (Model I).

**Additional file 2.** Receiver operating characteristic (ROC) curves for current tobacco users screening in students for both GYTS survey years.

**Additional file 3.** Receiver operating characteristic (ROC) curves for current tobacco users screening in students for pooled dataset.

## Data Availability

We have used publicly available datasets in this study which can be downloaded from the following links: Bangladesh - Global Youth Tobacco Survey 2007: https://extranet.who.int/ncdsmicrodata/index.php/catalog/767. Bangladesh - Global Youth Tobacco Survey 2013: https://extranet.who.int/ncdsmicrodata/index.php/catalog/232.

## Abbreviations

GYTS: Global Youth Tobacco Survey
CI: Confidence Interval
OR: Odds Ratio
TK: Bangladeshi Taka (currency)
