# Supplementary material for: Changes in prevalence, and factors associated with tobacco use among Bangladeshi school students: evidence from two nationally representative surveys
Source: BMC Public Health. 2021 Mar 23;21:579. doi: 10.1186/s12889-021-10623-0 (PMC7989242; doi:10.1186/s12889-021-10623-0)
Supplement: Supplementary file 1 — Additional file 1. The odds ratios for factors influencing tobacco use among adolescents for both survey years by binary logistic regression analysis (Model I). [file 12889_2021_10623_MOESM1_ESM.docx]

Additional file 1: Factors influencing tobacco use among adolescents for both survey years by binary logistic regression analysis (Model I).

| Covariates | 2007 | | 2013 | |
| --- | --- | --- | --- | --- |
|  | Odds Ratio  (95% CI) | P-value | Odds Ratio  (95% CI) | P-value |
| Age |  |  |  |  |
| Younger than 14 years | 1 |  | 1 |  |
| Equal to 14 years | 1.20 (0.58-2.51) | 0.614 | 1.73 (0.91-3.25) | 0.0892 |
| Older than 14 years | 1.67 (0.75-3.71) | 0.198 | 3.87 (2.21-6.78) | <.0001 |
| Sex |  |  |  |  |
| Female | 1 |  | 1 |  |
| Male | 1.64 (0.77-3.47) | 0.188 | 3.07 (1.51-6.25) | 0.0032 |
| Grade |  |  |  |  |
| Seven | 1 |  | 1 |  |
| Eight | 3.48 (0.90-13.49) | 0.070 |  |  |
| Above eight | 2.67 (0.62-11.50) | 0.179 |  |  |
| Average pocket money (per month) |  |  |  |  |
| Less than or equal to 160 TK | 1 |  |  |  |
| More than 160 TK | 4.50 (2.50-8.11) | < 0.000 |  |  |
| Less than or equal to 400 TK |  |  | 1 |  |
| Between 400 to 800 TK |  |  | 2.06 (0.48-8.89) | 0.3202 |
| More than or equal to 800 TK |  |  | 1.96 (0.26-14.59) | 0.4957 |
| Aware of the effect of passive smoking |  |  |  |  |
| Yes | 1 |  | 1 |  |
| No | 1.37 (0.50-3.76) | 0.534 | 0.61 (0.21-1.80) | 0.3569 |
| Favoured banning smoking at public places |  |  |  |  |
| Yes | 1 |  | 1 |  |
| No | 1.37 (0.64-2.91) | 0.403 | 2.05 (1.11-3.78) | 0.0228 |
| Average cost per packet cigarette (20 cigarettes) |  |  |  |  |
| More than 200 Tk |  |  | 1 |  |
| Less than or equal to 75 TK |  |  | 1.52 (0.64-3.59) | 0.3286 |
| Between 76 to 200 TK |  |  | 5.99 (3.13-11.46) | <.0001 |
| Health warning on tobacco products |  |  |  |  |
| Yes |  |  | 1.48(0.70-3.13) | 0.2895 |
| No |  |  | 1 |  |
| Being offered free tobacco products |  |  |  |  |
| Yes | 2.58 (1.32-5.06) | 0.008 | 0.46 (0.11-1.85) | 0.2603 |
| No | 1 |  | 1 |  |
| Taught about dangers of tobacco in class |  |  |  |  |
| Yes | 1 |  | 1 |  |
| No | 1.47 (0.87-2.49) | 0.148 |  |  |
| Noticed anyone smoking in the school |  |  |  |  |
| Yes |  |  | 2.24 (1.20-4.18) | 0.0137 |
| No |  |  |  |  |
| Exposed to tobacco smoke |  |  |  |  |
| Yes | 2.61 (1.01-6.75) | 0.048 | 5.05 (1.44-17.71) | 0.0134 |
| No | 1 |  | 1 |  |

CI: Confidence Interval. TK: Bangladeshi currency (1 USD ≈ 85 TK)
