# Supplementary material for: Changes in prevalence, and factors associated with tobacco use among Bangladeshi school students: evidence from two nationally representative surveys
Source: BMC Public Health. 2021 Mar 23;21:579. doi: 10.1186/s12889-021-10623-0 (PMC7989242; doi:10.1186/s12889-021-10623-0)

Additional file 2: Receiver operating characteristic (ROC) curves for current tobacco users screening in students for both GYTS survey years.


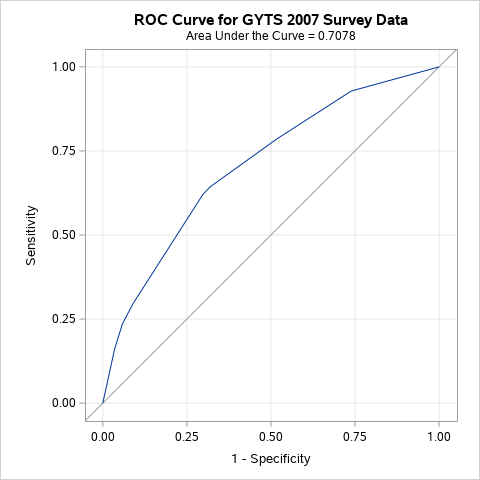

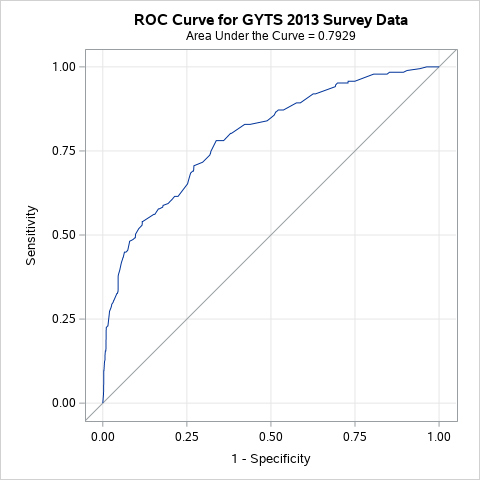

Supplement: Supplementary file 2 — Additional file 2. Receiver operating characteristic (ROC) curves for current tobacco users screening in students for both GYTS survey years. [file 12889_2021_10623_MOESM2_ESM.docx]
